# Effect of Different Head Hole Position on the Rotational Resistance and Stability of Orthodontic Miniscrews: A Three-Dimensional Finite Element Study

**DOI:** 10.3390/s21113798

**Published:** 2021-05-30

**Authors:** Jin-Young Choi, Min-Jung Kim, Seong-Hun Kim, Kyu-Rhim Chung, Gerald Nelson

**Affiliations:** 1 Department of Orthodontics, Kyung Hee University Dental Hospital, Seoul 02447, Korea; joyful.ortho@gmail.com; 2 Department of Convergence Medicine, Asan Medical Center, Asan Medical Institute of Convergence Science and 8 Technology, Seoul 02447, Korea; gracekim206@naver.com; 3 Department of Orthodontics, Graduate School of Dentistry, Kyung Hee University, Seoul 02447, Korea; speedor@hotmail.com; 4 Division of Orthodontics, Department of Orofacial Science, University of California, San Francisco, CA 94143, USA; gdnelson41@gmail.com

**Keywords:** miniscrew head hole, implantation angle, wire insertion angle

## Abstract

The orthodontic miniscrew is driven into bone in a clockwise direction. Counter-clockwise rotational force applied to the implanted miniscrew can degrade the stability. The purpose of this three-dimensional finite element study was to figure out the effect of shifting the miniscrew head hole position from the long axis. Two miniscrew models were developed, one with the head hole at the long axis and the other with an eccentric hole position. One degree of counter-clockwise rotation was applied to both groups, and the maximum Von-Mises stress and moment was measured under various wire insertion angles from −60° to +60°. All Von-Mises stress and moments increased with an increase in rotational angle or wire insertion angle. The increasing slope of moment in the eccentric hole group was significantly higher than that in the centric hole group. Although the maximum Von-Mises stress was higher in the eccentric hole group, the distribution of stress was not very different from the centric hole group. As the positive wire insertion angles generated a higher moment under a counter-clockwise rotational force, it is recommended to place the head hole considering the implanting direction of the miniscrew. Clinically, multidirectional and higher forces can be applied to the miniscrew with an eccentric head hole position.

## 1. Introduction

The temporary skeletal anchorage devices (TSADs) are an established powerful tool for efficient tooth movement. Reports indicate more effective correction of severe malocclusions and dentofacial discrepancies [1,2,3,4], such as en-masse retraction of anterior teeth [2,5], molar uprighting [6], maxillary expansion [7,8,9,10], skeletal open bite or deep bite correction [11,12], and anteroposterior discrepancy correction [13,14]. The successful use of TSADs in the clinic is highly related to their stability [15,16,17]. Several studies consider miniscrew design according to the requirements of clinical stability [18,19,20,21,22,23,24].

The orthodontic miniscrew is a common choice of TSAD for anchorage reinforce-ment [5,25], given the advantages of versatility, small in size, minimal surgical invasion, and low cost [26]. The mechanical stability of the miniscrew can be improved by design variations [19,20,27,28,29]. Based on a previous study, modifying the minis-crew’s length, diameter, and thread pitch or changing the body shape can affect stability [30,31,32,33]. Initially in the development of miniscrews, the focus was on microstructural modification of the miniscrew surface to improve osseointegration [34,35]. Then better osseointegration was obtained through macrostructure modifications to maximize the rotational resistance of orthodontic miniscrew [30].

The self-drilling miniscrew has sufficient primary stability, so that the orthodontic force can be immediately loaded [31,32]. Two factors reported to correlate with high stability and resistance to failure were insertion perpendicular to the alveolar bone surface and loading the orthodontic forces in the same direction as the long axis of the miniscrew [36,37]. Conversely, applying rotational, dynamic, or multidirectional forces may lead to instability. Unfortunately, placing the orthodontic miniscrew perfectly perpendicular to the alveolar bone surface is often impossible. In most clinical situations, the clinician has to avoid contact with vulnerable structures, necessitating placement at an optional angle [38,39,40]. Dependable rotational resistance is very important when using Biocreative Orthodontic Strategy (BOS) treatment, in which independent anterior traction is performed by applying only anterior brackets with a modified utility archwire to the head hole of the miniscrew, without applying posterior brackets [2,41]. Forces applied to the miniscrew for torque control of anterior retraction may act in an unscrewing direction [2]. When applying vertical forces to anterior teeth from an arch wire anchored to the hole in the head of a miniscrew, rotational stability is essential.

The objective of this study was to evaluate the rotational resistance in an orthodontic miniscrew with an eccentric head hole by measuring Von-Mises stresses and moments with various implantation angles. The null hypothesis was that there would not be a significant difference in rotational resistance between centric and eccentric head hole positions.

## 2. Materials and Methods

### 2.1. Finite Element Model

**Figure 1:**
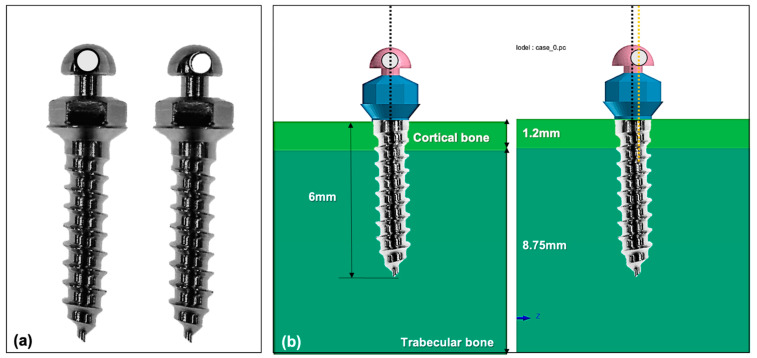
Two types of miniscrews based on the position of a hole in the head part: (**a**) the left one has a hole in the middle of the miniscrew’s head part (centric hole group), and the right one has a hole out of center of the miniscrew’s head part (eccentric hole group); (**b**) in the constructed model for this study, the two types of miniscrews were assumed to be placed totally into the alveolar bone. Thickness of the cortical bone was modeled at 1.2 mm, and the trabecular bone was thick enough to accept the whole length of the miniscrews. The left model is a miniscrew of the centric hole group and the right one is a miniscrew of the eccentric hole group.

The finite element models were constructed with 0.15 mm tetrahedral meshes. A virtual alveolar bone model was set up based on the cortical bone and trabecular bone using Visual-mesh software (version 7.0; ESI group, Paris, France). The thickness of cortical bone was set at 1.2 mm and the trabecular bone was set to be thick enough to accommodate the miniscrew. A model of the Bio-Action screw (Jin-Biomed co., Bucheon, Korea) was used for this study. The screw part maximum diameter was 1.6 mm, the overall length was 8.5 mm, and the thread area was 6.0 mm long. The Bio-Action screw consisted of an one component screw and head part, with three characteristics: (1) a narrowed thread at the uppermost part of the miniscrew, (2) a vertical notch at the middle third of screw part, and (3) a hole of 0.8 mm diameter at the head part of the miniscrew. To evaluate the effect of the hole position to the rotational resistance, two head parts were constructed: a control with the hole at the center of the head part (centric hole group), and an experimental head with the hole placed out of center (eccentric hole group) (Figure 1A). The material of the miniscrew was Ti-Grade-V-alloy (Ti-6Al-4V), the alloy combined α-β phases. The miniscrew was assumed to be placed completely into the alveolar bone without any exposure of thread part (Figure 1B). The wire inserted in the hole of head part was made of stainless steel, with a cross section of 0.017 by 0.025-inches. The algorithm of the augmented Lagrangian method was used with a surface-to-surface contact condition, and the coefficient of friction was set as 0.3. All the components were considered to be homogenous and isotropic. The material properties of each component are shown in Table 1.

### 2.2. Force Application

**Figure 2:**
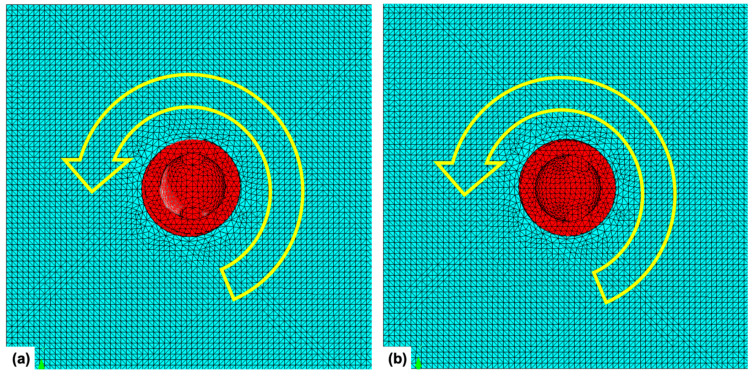
Direction of rotation applied to the miniscrew model: (**a**) on the top view of the miniscrew head part in the centric hole group, counterclockwise rotation was applied with the long axis of the miniscrew as a center of rotation; (**b**) same rotational direction (counterclockwise rotation) was applied to a miniscrew in the eccentric hole group.

**Figure 3:**
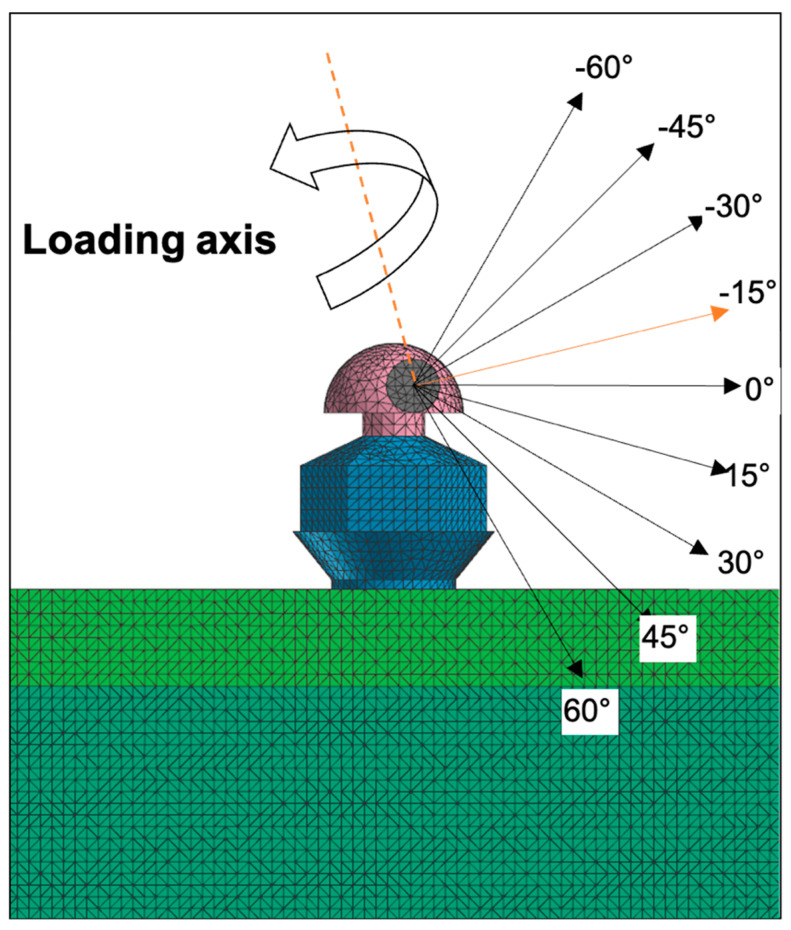
Miniscrews were assumed to be placed with various angulations on the alveolar bone surface. An archwire parallel to the alveolar bone and the occlusal plane passing through the hole of the angulated miniscrew forms corresponding angles with the long axis of the miniscrew. To make the same condition among the models, whole screw part was placed into the alveolar bone and the archwire was inserted with angles from −60° to +60° with an angle interval of 15°.

Because the miniscrews are inserted into the alveolar bone with a clockwise rotational direction, the rotational resistance can be measured with a counterclockwise rotational force (Figure 2). In the clinical situation, the wire is inserted into the hole of the head part, and the counterclockwise rotational force can be applied through the wire. Various insertion angles of the miniscrew were assumed in this study. Instead of using various angles of screw insertion, we simulated various screw insertion angles by varying the insertion angle of the arch wire into the head hole. The loading axis was perpendicular to the wire insertion angle, and the counterclockwise rotational force was loaded on the axis. The wire insertion angles were set from minus 60 degrees to plus 60 degrees at intervals of 15 degrees. Therefore, 9 conditions in each group were set up (Figure 3). Miniscrews were rotated progressively from 0 degree to 1 degree in all conditions. Maximum Von-Mises stress at the bone, and upper, middle, and apical third of the screw part was measured. The moment at the overall area, cortical bone, and trabecular bone was also calculated through the finite element analysis. The force loading simulations were performed by a Virtual Performance Solution (ver-sion 2008; ESI group, Paris, France) on a computer with Intel^®^ Xeon^®^ CPU E5-2680 @ 2.40 GHz X 28 core and the 128 GB RAM.

### 2.3. Statistical Analysis

We performed a generalized linear model to analyze the association between the moment required to unwind the miniscrew for 1° of counterclockwise rotation and insertion angles for each component (overall, cortical bone, and trabecular bone). In addition, an interaction effect analysis was used to figure out the difference of moment between the centric hole group and eccentric hole group. All statistical analyses were performed using SAS 9.4 program (SAS Institute Inc., Cary, NC, USA), and the significant value was 0.05.

## 3. Results

### 3.1. Von-Mises Stress

Maximum Von-Mises stress of the miniscrew in the eccentric hole group was higher than that in centric hole group at all the measured areas and all the wire insertion angles. The differences were the most at the upper third of the miniscrew, and the smaller differences were observed at the middle and apical thirds of the miniscrew. Almost all the values of Von-Mises stress were the lowest at 0° of wire insertion angle, and they increased as the insertion angle became steep (Table 2 and Table 3).

**Figure 4:**
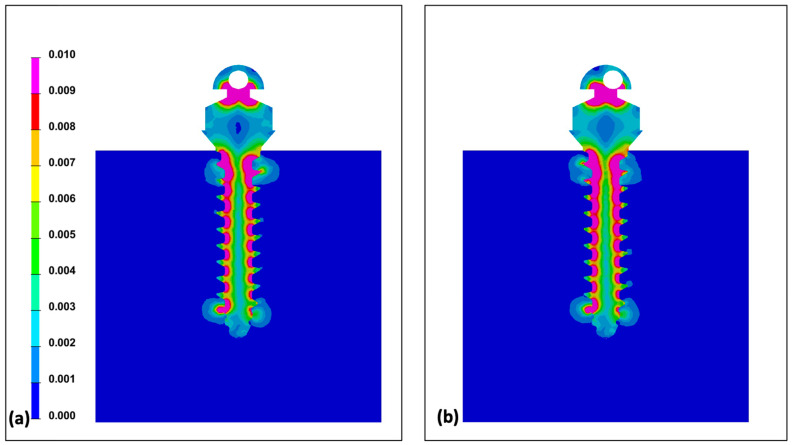
Distribution of Von-Mises stress under the condition of 1° counter-clockwise rotation of the miniscrew: (**a**) centric hole group; (**b**) eccentric hole group. Both groups showed relatively even distribution of Von-Mises stress.

**Figure 5:**
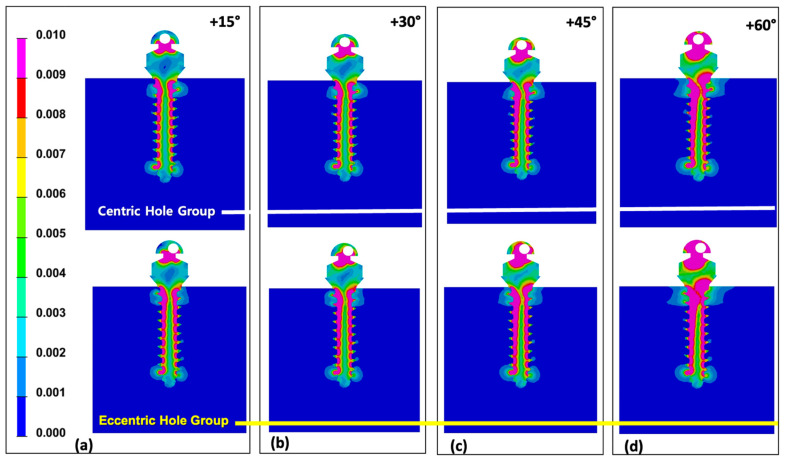
Distribution of Von-Mises stress under the condition of 1° counter-clockwise rotation of the miniscrew placed with various positive angles on the alveolar bone surface: (**a**) Von-Mises stress distribution of the miniscrew at +15° angulation in centric and eccentric hole groups; (**b**) Von-Mises stress distribution of the miniscrew at +30° angulation in centric and eccentric hole groups; (**c**) Von-Mises stress distribution of the miniscrew at +45° angulation in centric and eccentric hole groups; (**d**) Von-Mises stress distribution of the miniscrew at +60° angulation in centric and eccentric hole groups. In both groups, more stress was concentrated on the upper 1/3 as the angle increased.

**Figure 6:**
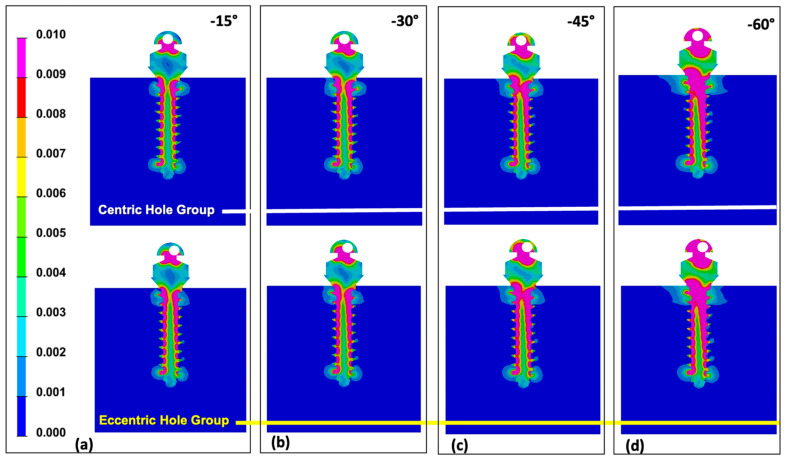
Distribution of maximum Von-Mises stress under the condition of 1° counter-clockwise rotation of the miniscrew placed with various negative angles on the alveolar bone surface: (**a**) Von-Mises stress distribution of the miniscrew at −15° angulation in centric and eccentric hole groups; (**b**) Von-Mises stress distribution of the miniscrew at −30° angulation in centric and eccentric hole groups; (**c**) Von-Mises stress distribution of the miniscrew at −45° angulation in centric and eccentric hole groups; (**d**) Von-Mises stress distribution of the miniscrew at −60° angulation in centric and eccentric hole groups. In both groups, more stress was concentrated on the upper 1/3 as the angle increased, and the stress distribution tendency was opposite to the result of the miniscrews implanted at positive angles.

**Figure 7:**
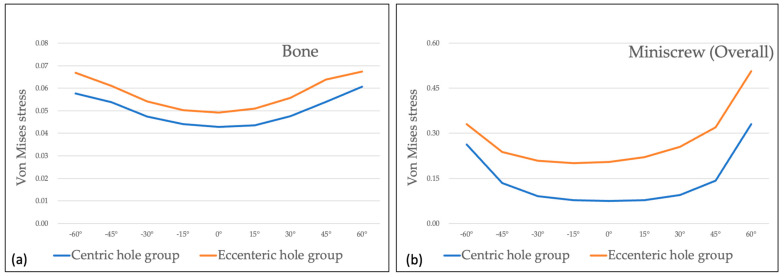
Von-Mises stress under 1° of counter-clockwise rotation at the alveolar bone and miniscrew at various angles: (**a**) Von-mises stress at the alveolar bone showing the higher stress in the centric hole group; (**b**) Von-Mises stress at the whole miniscrew showing the higher stress in the centric hole group and at positive angles.

**Figure 8:**
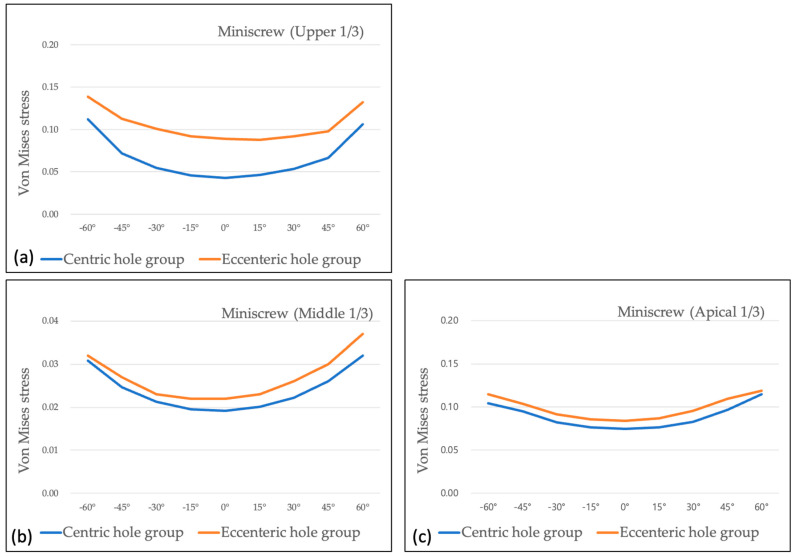
Maximum Von-Mises stress under 1° of counter-clockwise rotation at each part of the miniscrew: (**a**) Von-Mises stress at the upper 1/3 of the miniscrew showing relatively high value in the eccentric hole group and in the negative angles; (**b**) Von-Mises stress at the middle 1/3 of the miniscrew; (**c**) Von-Mises stress at the apical 1/3 of the miniscrew.

Comparing two groups at 0° of wire insertion angle, both centric and eccentric hole groups showed relatively even distribution despite of the higher Von-Mises stress value in the eccentric hole group (Figure 4). As the wire insertion angle increased either positively or negatively, it showed the concentrating tendency at the upper third of the miniscrew in both groups (Figure 5 and Figure 6). Fairly symmetric charts with the center at 0° could be drawn except the Von-Mises stress in overall miniscrew parts (Figure 7 and Figure 8). The overall Von-Mises stress generated at the miniscrew was higher when the wire was inserted in the positive angle than when the wire was inserted in the negative angle, especially at 60 degrees (Figure 7b).

### 3.2. Moment Required to Unwind the Miniscrew

**Figure 9:**
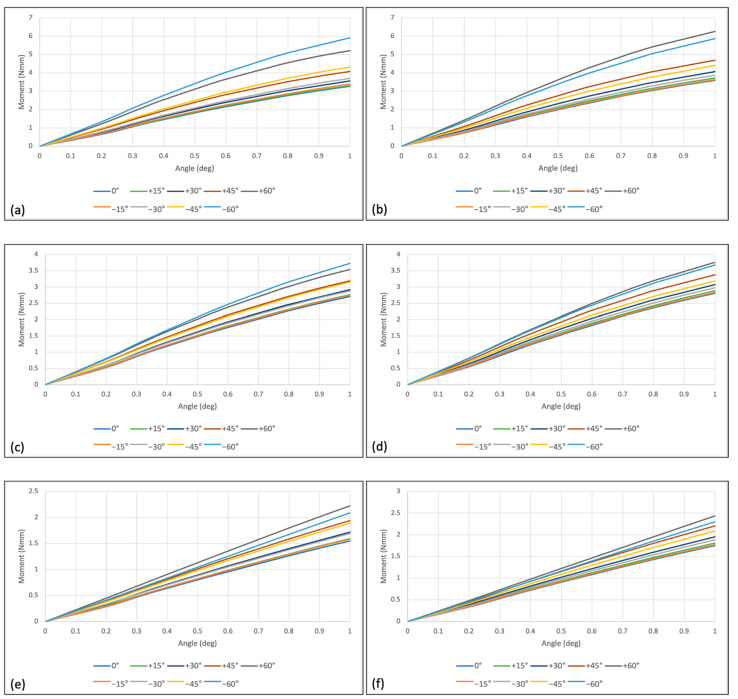
Moment required to unwind the miniscrew for 1° in counter-clockwise direction in centrical and eccentric hole groups: (**a**) overall moment in the centric hole group; (**b**) overall moment in the eccentric hole group; (**c**) moment at the cortical bone in the centric hole group; (**d**) moment at the cortical bone in the eccentric hole group; (**e**) moment at the trabecular bone in the centric hole group; (**f**) moment at the trabecular bone in the eccentric hole group.

All the moments at cortical and trabecular bone, and the overall moments generated in the condition of 1° counterclockwise rotation in both groups were increased as the rotational angle increased from 0° to 1° (Figure 9). Regardless of the position of the hole at the head part, all models showed significant higher moment at cortical bone level than that at trabecular bone level. The overall moment, which was considered as a combination of the moment at the cortical bone and the moment at the trabecular bone, showed much higher value.

The increasing tendency of the moment required to unwind the miniscrew for 1° of counter-clockwise rotation was confirmed by generalized linear model. In both positive and negative wire insertion angles, moment increased as the insertion angle increased at the overall, cortical bone, and trabecular bone areas. While the moments of the eccentric hole group were higher than the centric hole group with the positive wire insertion angle, when it comes to the negative wire insertion angle, moments of both centric and eccentric hole groups were comparable (Table 4 and Table 5).

As the miniscrew’s counter-clockwise rotation angle increased from 0° to 1°, the moments at overall, cortical bone, and trabecular areas all increased with statistical significance. It was confirmed by the generalized linear model with an adjustment of wire insertion angles (Table 6).

With an interaction effect analysis, differences of the moment between centric hole group and eccentric hole groups were also calculated. When the direction (positive/negative) and the counter-clockwise rotation were adjusted, the increasing amount of moment with the change of wire insertion angle in the centric hole group and eccentric hole group was not significantly different. On the other hand, when the direction and wire insertion angle were adjusted, the increasing amount of moment with the change of rotation angle was higher in the eccentric hole group than in the centric hole group (Table 7).

## 4. Discussion

Using miniscrew anchorage does not guarantee successful treatment in all clinical applications. The stability of the miniscrew is a basic prerequisite for success. Previous reports have presented treatment using a tube type miniplate (C-tube plate), anchored by multiple anchoring screws, or by a surface treated mini-implant (C-implant). While these methods avoid concern of counter-clockwise loosening of the TSAD, they have disadvantages of complexity and difficulty in manufacturing, and a need for pre-drilling and flap surgery, which is not preferred [42,43]. As a better alternative, we have designed a new orthodontic miniscrew called the Bio-Action screw. The goal was to increase rotational resistance by eccentrically positioning the hole in the head of the screw. Displacing the hole position from the long axis can provide a stronger rotational resistance compared to the head hole at the long axis of the miniscrew. In this study, a finite element model was established to analyze the stress distribution of two head hole positions and the maximum Von-Mises stresses and moments of the orthodontic wire inserted into the head hole with different implantation angles. Distribution of Von-Mises stress was not quite different between the centric hole group and eccentric hole group, which means there was no worrisome stress-concentrated area on the miniscrew or alveolar bone. As either wire insertion angles or counter-clockwise rotational angle of the miniscrew increased, the moment also increased with the statistical significance. The slope of moment increase from 0° to 1° counter-clockwise rotation of the miniscrew was higher in the eccentric hole group than in the centric hole group. Because the maximum Von-Mises stress and the moment required to unwind the miniscrew were all higher in the eccentric hole group than in the centric hole group, the null hypothesis was rejected in our study.

From a mechanical point of view, the implantation angle of a miniscrew is one of affecting factors for stability. Jasmine et al. [16] analyzed the stress distribution on alveolar bone and miniscrews with different implantation angles using the three-dimensional finite element analysis method. They observed more stability when the mini-implant is perpendicular to the long axis of the teeth. Contrarily, Liu et al. [44] reported that mini-implant insertion with an oblique angle increased primary stability. Popa et al. [15] used the finite element analysis to reveal that the cortical bone stress showed lowest value and highest stability when the mini-implant had a 30° implantation angle, and when the cortical bone thickness was 1 mm. Meanwhile, when the cortical bone thickness was 2 mm, the cortical bone stress had the lowest value with an implantation angle of 90°. However, results of the finite element model highly depend on the model developed. We evaluated the rotational resistance of the counterclockwise rotational force applied to the miniscrew. This may be the reason why our results showed disagreement with tendency of implantation angle and stress distribution previously reported. Figure 7 shows that stress was increased along with the implantation angle. A higher Von-Mises stress was observed in the miniscrew with the eccentric head hole in all cases. We assumed that the miniscrew was rotated for 1° in the counter-clockwise direction, and the eccentric hole miniscrew had better rotational resistance than the centric hole miniscrew. Since the miniscrew rotates along the axis of rotation, the eccentric hole position is slightly off the long axis of the miniscrew and generated different stress distributions (Figure 4, Figure 5 and Figure 6).

The stability of a miniscrew is affected by stress applied to the surrounding bone [9,31,38,45,46,47]. In this study, we used a finite element analysis model to measure the stress values on the surrounding bone and miniscrew at each part. Ghorbanyjavadpour et al. [48] compared stress distribution between tapered and cylindrical miniscrews during implantation and removal. They observed the highest stresses in both the miniscrew and bone. The stress decreased when miniscrew entered the cancellous bone, and the stress was concentrated around the miniscrew neck at removal. Their findings might help to explain why our results showed a concentrating tendency at the upper third of the miniscrew in both groups, in spite of changing the wire insertion angle either positively or negatively. In agreement with the previous studies, whether the head hole positioned centric or eccentric, all moment measurements had significantly higher moments at the cortical bone level than that at the trabecular bone level [15,16,38,49,50]. Because most stress occurs during implantation or removal at the cortical bone [48], the condition of cortical bone should be good and the mini screw design appropriate. Excessive concentration to a specific region would be undesirable. In this study, the stress concentration level was not significantly different between the centric hole group and eccentric hole group, so the change of hole position does not increase risks of miniscrew fracture or bone damage.

**Figure 10:**
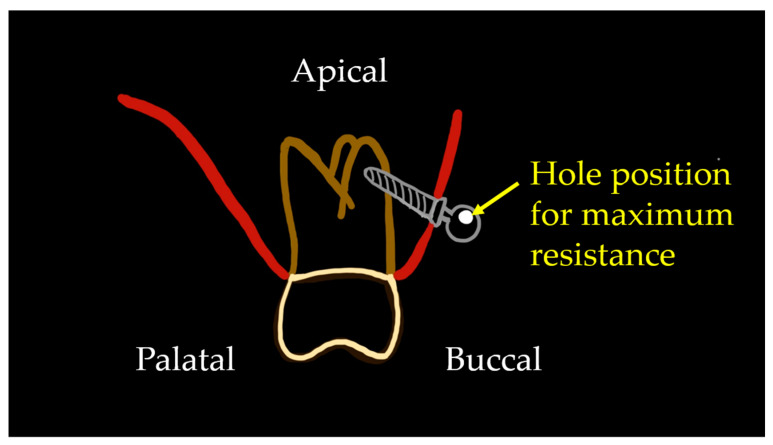
Suggestion of an ideal head hole position for maximum resistance to counter-clockwise rotational force. When the miniscrew tip directed the apical side with an angulation to the alveolar bone surface, the hole positioned on the upper (apical) side of the head would generate the maximum resistance to the rotational force.

The orthodontic miniscrew is implanted with clockwise rotation. Gracco et al. [19] compared four miniscrews with different thread shapes to determine the effects of variations in thread shape on the axial pullout strength of orthodontic miniscrews. Four experimental miniscrews were compared to a control miniscrew. Results showed the buttress reverse thread shape provided the greatest pullout strength value, which was to 192.8 N. Considering this evidence, the miniscrew used in our study provides the same benefit of the buttress reverse thread shape for improved stability. Interestingly, the maximum Von-Mises stress in the eccentric hole group showed a lower value when the wire was placed at the positive angle than when the wire was inserted at the negative angle, especially at +/− 60 degrees. In addition, the miniscrew with centric head hole at a positive implantation angle showed higher moment values than at negative implantation angles. The threads of screw have an angulation with the long axis of the miniscrew and so there is an interaction between the wire insertion angle and the direction of the threads. This factor might affect the different Von-Mises stress and moments measured between the models with positive and negative wire insertion angles. Putting the results together, the positive wire insertion angle was more favorable when a counter-clockwise rotational force was applied. Clinically, to achieve higher resistance to the counter-clockwise rotational force, the wire insertion angle should be positive considering the force direction in Figure 3. It would be desirable to adjust the eccentric hole position to the direction of the tip of the miniscrew. For example, when the miniscrew is planned to be placed on the maxillary buccal side with the angulation to the alveolar bone surface so that the tip of the miniscrew directed the upper (apical) side, the eccentric hole should be positioned to be parallel to the occlusal plane and to be on the upper side of the head, in order to resist the counter-clockwise directional force (Figure 10).

The hole at the miniscrew head part can be used in several purposes. Kim et al. suggested the archwire inserted into the head hole of orthodontic miniscrew instead of the fixed appliances on the posterior teeth in the extraction cases [51]. Torque of the anterior teeth can be controlled by an adjustment of the wire such as a v-bend [2]. An auxiliary wire segment passing through the hole with an open coil spring also plays a role in pushing molars distally [52,53]. Because the miniscrew used in these manners should endure multidirectional and strong forces, the stability of miniscrew has been considered as the main point for a successful application.

Most orthodontic miniscrew stability studies have studied length, thread, diameter, and surface treatment. In the previous finite element study [54], the screw part was modified with a vertical notch at the middle of screw and a narrowed uppermost area of the screw, and the power of these screw modifications on the stability was confirmed. The new modifications of the miniscrew used in this study demonstrated the increase in stability. In regard to surface treated orthodontic TSADs, adding the eccentric head hole position could further increase the rotational resistance, which could be useful in more demanding force applications. Because our study results were based on computational calculation with the three-dimensional virtual model, the actual reaction might be different to the expected result. Although the model for finite element analysis has a limitation that it is impossible to reproduce the actual conditions perfectly, the finite element analysis substitutes for in vivo test because only the factor of interest can be modified to figure out the effect of it leaving the other factors are fixed, as Chieruzzi et al. suggested in their study [55]. On the other hand, the shoulder part of the miniscrew model was supposed to be placed exactly at the level of bone surface. In the actual condition, the miniscrew is impossible to be inserted evenly, especially it is placed with an angulation. Clinical outcomes with the modified miniscrews used in this study should be compared and analyzed in the future.

## 5. Conclusions

Modification of the hole on the miniscrew head part from a centric to eccentric position raised the moment required to unwind the miniscrew in counter-clockwise direction.

As the insertion angles increased either positively or negatively, the moment also increased with the statistical significance.

The miniscrew with an eccentric head hole in addition to the macrostructural modification of screw part can be an alternative to the conventional miniscrew, when the multidirectional and strong forces are required.

It is recommended that the clinicians should place the miniscrew with its head hole positioned on the side which the screw tip directed.

## 6. Patents

Jin biomed company owns a patent of Bio-Action screw design (patent no. 10-2019-0102799).

## Figures and Tables

**Table 1:** The material properties used for the finite element analysis.

	Young’s Modulus (GPa)	Poisson’s Ratio
Cortical bone	13.7	0.3
Trabecular bone	1.37	0.3
Titanium alloy	110	0.35

**Table 2:** Maximum Von-Mises stress (GPa) at the bone and each part of the orthodontic miniscrew according to the position of the hole in the head part and the positive wire insertion angles.

	Group	0°	+15°	+30°	+45°	+60°
Bone	Centric	0.043	0.044	0.048	0.054	0.061
	Eccentric	0.049	0.051	0.056	0.064	0.067
Miniscrew (Overall)	Centric	0.075	0.077	0.095	0.142	0.331
	Eccentric	0.205	0.221	0.254	0.319	0.506
Miniscrew (Upper 1/3)	Centric	0.043	0.047	0.054	0.067	0.106
	Eccentric	0.089	0.088	0.092	0.098	0.132
Miniscrew (Middle 1/3)	Centric	0.019	0.020	0.022	0.026	0.032
	Eccentric	0.022	0.023	0.026	0.030	0.037
Miniscrew (Apical 1/3)	Centric	0.075	0.077	0.083	0.097	0.115
	Eccentric	0.084	0.087	0.096	0.110	0.119

**Table 3:** Maximum Von-Mises stress (GPa) at the bone and each part of the orthodontic miniscrew according to the position of the hole in the head part and the negative wire insertion angles.

	Group	0°	−15°	−30°	−45°	−60°
Bone	Centric	0.043	0.044	0.048	0.054	0.058
	Eccentric	0.049	0.050	0.054	0.061	0.067
Miniscrew (Overall)	Centric	0.075	0.077	0.090	0.134	0.263
	Eccentric	0.205	0.201	0.209	0.238	0.331
Miniscrew (Upper 1/3)	Centric	0.043	0.046	0.055	0.072	0.112
	Eccentric	0.089	0.092	0.101	0.113	0.139
Miniscrew (Middle 1/3)	Centric	0.019	0.020	0.021	0.025	0.031
	Eccentric	0.022	0.022	0.023	0.027	0.032
Miniscrew (Apical 1/3)	Centric	0.075	0.077	0.083	0.096	0.104
	Eccentric	0.084	0.086	0.092	0.104	0.115

**Table 4:** Moment (Nmm) required to unwind the miniscrew for 1° of counter-clockwise rotation at each component of finite element model according to the position of the hole at the head part and the positive wire insertion angles.

	Group	Wire Insertion Angle					Generalized Linear Model			
		0°	+15°	+30°	+45°	+60°	Slope	CI		*p*-Value
Overall	Centric	3.268	3.307	3.557	4.078	5.207	0.336	0.299	0.372	<0.0001
	Eccentric	3.591	3.713	4.061	4.684	6.255	0.442	0.395	0.490	<0.0001
Cortical bone	Centric	2.711	2.768	2.923	3.194	3.543	0.149	0.134	0.163	<0.0001
	Eccentric	2.809	2.885	3.074	3.373	3.754	0.167	0.152	0.183	<0.0001
Trabecular bone	Centric	1.550	1.595	1.718	1.940	2.225	0.102	0.093	0.112	<0.0001
	Eccentric	1.749	1.808	1.955	2.201	2.434	0.106	0.096	0.115	<0.0001

**Table 5:** Moment (Nmm) required to unwind the miniscrew for 1° of counter-clockwise rotation at each component of finite element model according to the position of the hole at the head part and the negative wire insertion angles.

	Group	Wire Insertion Angle					Generalized Linear Model			
		0°	−15°	−30°	−45°	−60°	Slope	CI		*p*-Value
Overall	Centric	3.268	3.388	3.705	4.313	5.908	0.423	0.377	0.470	<0.0001
	Eccentric	3.591	3.629	3.862	4.408	5.869	0.371	0.326	0.416	<0.0001
Cortical bone	Centric	2.711	2.748	2.881	3.152	3.732	0.165	0.147	0.183	<0.0001
	Eccentric	2.809	2.840	2.978	3.190	3.684	0.146	0.130	0.162	<0.0001
Trabecular bone	Centric	1.550	1.582	1.691	1.891	2.089	0.080	0.073	0.088	<0.0001
	Eccentric	1.749	1.774	1.885	2.085	2.297	0.085	0.077	0.093	<0.0001

**Table 6:** Moment (Nmm) required to unwind the miniscrew for 0° to 1° of counter-clockwise rotation in 0.2° interval at each component of finite element model according to the position of the hole at the head part of the miniscrew placed with 60° angulation.

	Group	Counter-Clockwise Rotation Angle					Generalized Linear Model			
		0.2°	0.4°	0.6°	0.8°	1.0°	Slope	CI		*p*-Value
Overall	Centric	1.282	2.655	3.841	4.822	5.558	4.017	3.864	4.171	<0.0001
	Eccentric	1.375	2.842	4.141	5.233	6.062	4.387	4.221	4.553	<0.0001
Cortical bone	Centric	0.799	1.660	2.428	3.093	3.637	3.043	2.984	3.103	<0.0001
	Eccentric	0.818	1.683	2.471	3.158	3.719	3.144	3.086	3.202	<0.0001
Trabecular bone	Centric	0.432	0.871	1.306	1.738	2.157	1.811	1.779	1.843	<0.0001
	Eccentric	0.470	0.953	1.430	1.903	2.366	2.022	1.990	2.055	<0.0001

**Table 7:** Differences of moment between the centric hole group and eccentric hole group with the change of wire insertion angle and counter-clockwise rotation angle.

Variable	Area	Generalized Linear Model			
		Slope	CI		*p*-Value
Wire insertion angle	Overall	0.030	−0.016	0.076	0.1995
	Cortical bone	−0.001	−0.018	0.015	0.8621
	Trabecular bone	0.004	−0.006	0.014	0.4374
Rotation angle	Overall	0.370	0.138	0.602	0.0019
	Cortical bone	0.101	0.017	0.184	0.0187
	Trabecular bone	0.211	0.166	0.256	<0.0001

## Data Availability

Data sharing not applicable.
